# Tetra­kis(μ-penta­fluoro­benzoato-κ^2^ *O*:*O*′)bis­[(tetra­hydro­furan-κ*O*)molybdenum(II)]

**DOI:** 10.1107/S1600536811033411

**Published:** 2011-08-27

**Authors:** Li-Juan Han

**Affiliations:** a Department of Chemistry, Tongji University, Shanghai 200092, People’s Republic of China

## Abstract

In the asymmetric unit of the title compound, [Mo_2_(C_7_F_5_O_2_)_4_(C_4_H_8_O)_2_], two independent half-mol­ecules are present, which are completed by a crystallographically imposed center of inversion between the individual Mo atoms. In each mol­ecule, four penta­fluoro­benzoate anions bridge the quadruply bonded Mo_2_^4+^ unit that is, in addition, axially coordinated by two O atoms of tetra­hydro­furan (THF) mol­ecules. In the two independent mol­ecules, the mean Mo—Mo bond length is 2.110 Å. Since the THF mol­ecules are equally disordered over two sets of sites, there are four different Mo—O distances in both half-mol­ecules with an overall mean of 2.542 Å. A zigzag chain is formed by π–π stacking inter­actions between penta­fluoro­phenyl rings, indicated by a centroid–centroid distance of 3.7054 (11) Å and a centroid-to-plane distance of 3.4169 (3) Å. The extension of the unit gives a three-dimensional network structure with the THF mol­ecules located in the voids.

## Related literature

For phen­yl–phenyl π–π stacking, see: Carroll *et al.* (2008 ▶); Gung *et al.* (2005 ▶); McNeil *et al.* (2006 ▶); Sui & Glaser (2006 ▶). For phen­yl–perfluoro­phenyl π–π stacking, see: Vangala *et al.* (2002 ▶); Woody *et al.* (2007 ▶); Xu *et al.* (2008 ▶); Zhu *et al.* (2005 ▶). For perfluoro­phen­yl–perfluoro­phenyl π–π stacking, see: Adams *et al.* (2001 ▶); Hair *et al.* (2003 ▶); Liu *et al.* (2003 ▶). For torsion angles about penta­fluoro­benzonate anions, see: Reddy *et al.* (2004 ▶); Bach *et al.* (2001 ▶).
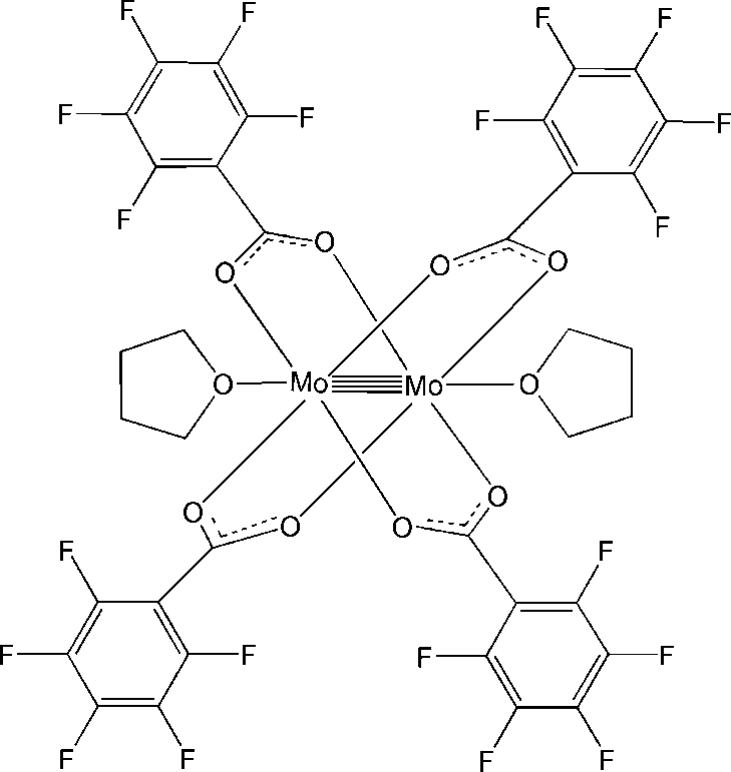


## Experimental

### 

#### Crystal data

[Mo_2_(C_7_F_5_O_2_)_4_(C_4_H_8_O)_2_]
*M* *_r_* = 1180.37
Triclinic, 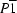

*a* = 11.101 (4) Å
*b* = 12.113 (4) Å
*c* = 15.741 (5) Å
α = 75.657 (4)°
β = 80.658 (4)°
γ = 86.813 (2)°
*V* = 2023.3 (11) Å^3^
*Z* = 2
Mo *K*α radiation
μ = 0.77 mm^−1^
*T* = 293 K
0.25 × 0.20 × 0.20 mm

#### Data collection

Bruker APEXII CCD diffractometer
Absorption correction: multi-scan (*SADABS*; Sheldrick, 2004 ▶) *T*_min_ = 0.825, *T*_max_ = 0.857
10491 measured reflections
6988 independent reflections
5663 reflections with *I* > 2σ(*I*)
*R*_int_ = 0.015

#### Refinement

*R*[*F* ^2^ > 2σ(*F* ^2^)] = 0.034
*wR*(*F* ^2^) = 0.089
*S* = 1.02
6988 reflections
648 parameters
5 restraints
H-atom parameters constrained
Δρ_max_ = 0.54 e Å^−3^
Δρ_min_ = −0.45 e Å^−3^

### 

Data collection: *APEX2* (Bruker, 2004 ▶); cell refinement: *SAINT-Plus* (Bruker, 2001 ▶); data reduction: *SAINT-Plus*; program(s) used to solve structure: *SHELXS97* (Sheldrick, 2008 ▶); program(s) used to refine structure: *SHELXL97* (Sheldrick, 2008 ▶); molecular graphics: *XP* in *SHELXTL* (Sheldrick, 2008 ▶) and *DIAMOND* (Brandenburg, 1999 ▶); software used to prepare material for publication: *SHELXL97*.

## Supplementary Material

Crystal structure: contains datablock(s) global, I. DOI: 10.1107/S1600536811033411/wm2516sup1.cif

Structure factors: contains datablock(s) I. DOI: 10.1107/S1600536811033411/wm2516Isup2.hkl

Additional supplementary materials:  crystallographic information; 3D view; checkCIF report

## Figures and Tables

**Table 1:** Selected bond lengths (Å)

Mo1—O3^i^	2.096 (2)
Mo1—Mo1^i^	2.1090 (7)
Mo1—O1^i^	2.111 (2)
Mo1—O2	2.116 (2)
Mo1—O4	2.118 (2)
Mo1—O2*S*	2.530 (9)
Mo1—O1*S*	2.543 (12)
Mo2—O6	2.106 (3)
Mo2—O7^ii^	2.108 (2)
Mo2—Mo2^ii^	2.1101 (8)
Mo2—O5	2.113 (3)
Mo2—O8	2.118 (2)
Mo2—O4*S*	2.544 (14)
Mo2—O3*S*	2.552 (4)

Symmetry codes: (i) 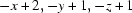; (ii) 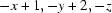.

**Fig. 1.**
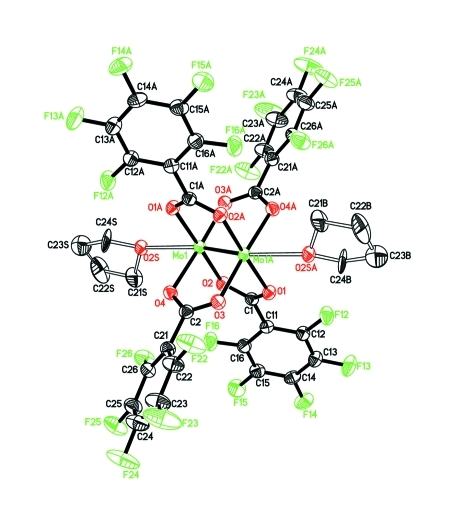
Molecular structure of one of the two independent molecules drawn with displacement ellipsoids at the 30% probability level. All hydrogen atoms and the second set of the disordered THF molecule have been omitted for clarity. [Symmetry code (A): -x + 2, -y + 1, -z + 1.]

**Fig. 2.**
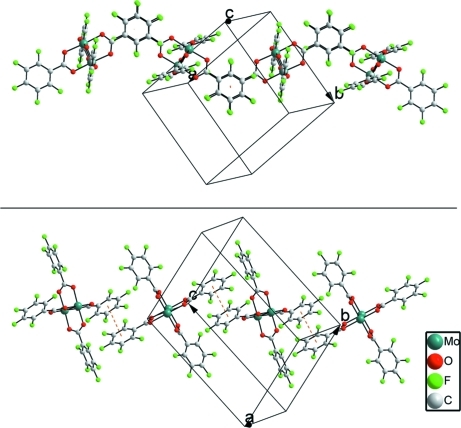
Part of a zigzag one-dimensional linear chain formed by the π–π stacking viewed in two different directions. All THF molecules have been omitted for clarity

**Fig. 3.**
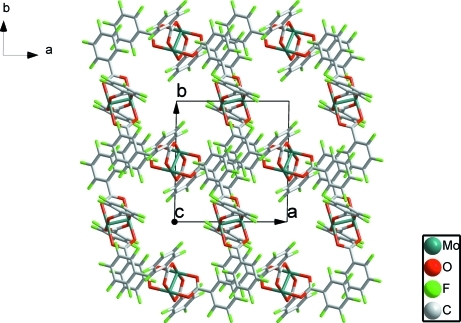
Part of three-dimensional network viewed along c axis. All THF molecules have been omitted for clarity

**Fig. 4.**
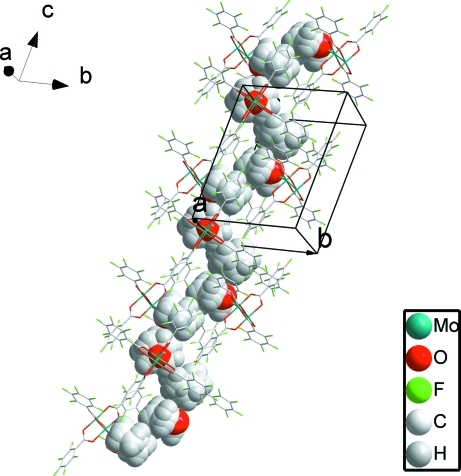
Part of the network with THF molecules (displayed in the spacefill mode) in the voids.

## supplementary crystallographic information

### Comment

Interactions between aromatic rings *via*π–π stacking are the basis of many phenomena with respect to organic material science and biological chemistry. In the last ten years, phenyl–phenyl (Carroll *et al.*, 2008; Gung *et al.*, 2005; McNeil *et al.*, 2006; Sui & Glaser, 2006) and phenyl–perfluorophenyl (Vangala *et al.*, 2002; Woody *et al.*, 2007; Xu *et al.*, 2008; Zhu *et al.*, 2005) interactions have been widely discussed in terms of supermolecular chemistry, but there appear to be few examples about the interactions between perfluorophenyl rings (Adams *et al.*, 2001; Hair *et al.*, 2003; Liu *et al.*, 2003). In the present study, π–π stacking interactions between the perfluorophenyl rings of the quadruply bonded dimetal paddlewheel molecule were investigated.

In the title compound, [Mo_2_(OOCC_6_F_5_)_4_(C_4_H_8_O)_2_], two independent half-molecules are present in the asymmetric unit, which are completed by a crystallographically imposed center of inversion between each of the Mo atoms. Each [Mo_2_(OOCC_6_F_5_)_4_(C_4_H_8_O)_2_] molecule has a paddle-wheel-type structure. There are four pentafluoro-benzoate (OOCC_6_F_5_) groups surrounding the quadruply bonded Mo_2_^4+^ unit, that is additionally axially coordinated by two oxygen atoms of THF molecules. In the two independent molecules, the Mo—Mo bond lengths are 2.1090 Å and 2.1101 Å, with a mean of 2.110 Å. Since the THF molecules are equally disordered over two sets of sites, there are four Mo–O distances to the THF molecules in both half-molecules (Table 1), with an overall mean of 2.542 Å. The molecular structure of one of the two molecules is shown in Fig. 1. The torsion angles between the C_6_F_5_ group and the connected chelating ring (Mo_2_OCO) range from -28.1 (5) ° to 41.7 (5) ° because of the O···F repulsion within the pentafluoro-benzonate anion (Reddy *et al.*, 2004; Bach *et al.*, 2001).

A zigzag chain (Fig. 2) is formed by π–π stacking interactions between pentafluorophenyl rings [indicated by the center-to-center distance of 3.7054 (11) Å and center-to-plane distance of 3.4169 (3) Å between two pentafluorophenyl rings]. In the crystal, the extension of the unit gives a three-dimensional network structure (Fig. 3) with the THF molecules situated in the voids (Fig. 4).

### Experimental

Mo(CO)_6_ (0.422 g, 1.60 mmol), pentafluorobenzoic acid (0.890 g, 4.20 mmol) and THF (2 ml) were mixed in 1,2-dichlorobenzene (6 ml) in a Schlenk flask equipped with a condenser. The mixture was then heated at 453 K for 24 h, during which a dark solution developed. After the reaction was cooled to room temperature, the THF was evaporated under reduced pressure, giving a yellow suspension. The mother liquor was decanted and the solid was washed first with dichloromethane (*ca* 8 × 2 ml), then with hexanes (*ca* 10 × 2 ml). The yellow solid product Mo_2_(O_2_CC_6_F_5_)_4_ was dried *in vacuo*. Yield: 0.68 g (82%). Anal. calcd. for C_28_O_8_F_20_Mo_2_: C,32.46; Found: C, 32.21. 0.207 g yellow powder of Mo_2_(O_2_CC_6_F_5_)_4_ was dissolved in 10 ml THF in a Schlenk tube and the solution was carefully layered with 30 ml hexanes. Yellow block-shaped crystals of the THF adduct formed after one week. Yield: 0.104 g (50%).

### Refinement

All H atoms were placed in idealized positions (C—H = 0.93–0.97 Å, O—H = 0.82 Å and refined as riding atoms with *U*_iso_(H) = 1.2*U*_eq_(C) and with *U*_iso_(H) = 1.5*U*_eq_(O).

### Crystal data

**Table:**

[Mo_2_(C_7_F_5_O_2_)_4_(C_4_H_8_O)_2_]	*Z* = 2
*M**_r_* = 1180.37	*F*(000) = 1152
Triclinic, *P*1	*D*_x_ = 1.937 Mg m^−^^3^
Hall symbol: -P 1	Mo *K*α radiation, λ = 0.71073 Å
*a* = 11.101 (4) Å	Cell parameters from 5431 reflections
*b* = 12.113 (4) Å	θ = 2.4–27.2°
*c* = 15.741 (5) Å	µ = 0.77 mm^−^^1^
α = 75.657 (4)°	*T* = 293 K
β = 80.658 (4)°	Block, yellow
γ = 86.813 (2)°	0.25 × 0.20 × 0.20 mm
*V* = 2023.3 (11) Å^3^	

### Data collection

**Table:**

Bruker APEXII CCD diffractometer	6988 independent reflections
Radiation source: fine-focus sealed tube	5663 reflections with *I* > 2σ(*I*)
graphite	*R*_int_ = 0.015
ω scans	θ_max_ = 25.0°, θ_min_ = 2.4°
Absorption correction: multi-scan (*SADABS*; Sheldrick, 2004)	*h* = −12→13
*T*_min_ = 0.825, *T*_max_ = 0.857	*k* = −12→14
10491 measured reflections	*l* = −14→18

### Refinement

**Table:**

Refinement on *F*^2^	Primary atom site location: structure-invariant direct methods
Least-squares matrix: full	Secondary atom site location: difference Fourier map
*R*[*F*^2^ > 2σ(*F*^2^)] = 0.034	Hydrogen site location: inferred from neighbouring sites
*wR*(*F*^2^) = 0.089	H-atom parameters constrained
*S* = 1.02	*w* = 1/[σ^2^(*F*_o_^2^) + (0.0456*P*)^2^ + 1.0297*P*] where *P* = (*F*_o_^2^ + 2*F*_c_^2^)/3
6988 reflections	(Δ/σ)_max_ = 0.001
648 parameters	Δρ_max_ = 0.54 e Å^−^^3^
5 restraints	Δρ_min_ = −0.45 e Å^−^^3^

### Special details

**Table:**

Geometry. All e.s.d.'s (except the e.s.d. in the dihedral angle between two l.s. planes) are estimated using the full covariance matrix. The cell e.s.d.'s are taken into account individually in the estimation of e.s.d.'s in distances, angles and torsion angles; correlations between e.s.d.'s in cell parameters are only used when they are defined by crystal symmetry. An approximate (isotropic) treatment of cell e.s.d.'s is used for estimating e.s.d.'s involving l.s. planes.
Refinement. Refinement of *F*^2^ against ALL reflections. The weighted *R*-factor *wR* and goodness of fit *S* are based on *F*^2^, conventional *R*-factors *R* are based on *F*, with *F* set to zero for negative *F*^2^. The threshold expression of *F*^2^ > σ(*F*^2^) is used only for calculating *R*-factors(gt) *etc*. and is not relevant to the choice of reflections for refinement. *R*-factors based on *F*^2^ are statistically about twice as large as those based on *F*, and *R*- factors based on ALL data will be even larger.

### Fractional atomic coordinates and isotropic or equivalent isotropic displacement parameters (Å^2^)

**Table:**

	*x*	*y*	*z*	*U*_iso_*/*U*_eq_	Occ. (<1)
Mo1	0.96580 (2)	0.42096 (2)	0.539073 (17)	0.03647 (8)	
Mo2	0.40712 (3)	0.98024 (2)	0.02261 (2)	0.04568 (9)	
O1	0.86392 (19)	0.62518 (17)	0.41781 (14)	0.0423 (5)	
O2	0.79076 (19)	0.45892 (17)	0.50142 (14)	0.0421 (5)	
O3	1.0837 (2)	0.51557 (18)	0.34755 (14)	0.0443 (5)	
O4	1.0128 (2)	0.34745 (17)	0.42904 (14)	0.0442 (5)	
O5	0.3922 (2)	0.9317 (2)	−0.09523 (16)	0.0527 (6)	
O6	0.4112 (2)	1.0262 (2)	0.14266 (16)	0.0520 (6)	
O7	0.6502 (2)	0.85278 (19)	0.03440 (16)	0.0504 (6)	
O8	0.4543 (2)	0.81023 (18)	0.08272 (16)	0.0496 (6)	
F12	0.6836 (2)	0.77847 (16)	0.38710 (17)	0.0683 (6)	
F13	0.4803 (2)	0.82323 (19)	0.31548 (19)	0.0835 (8)	
F14	0.3450 (2)	0.6530 (2)	0.29962 (17)	0.0794 (7)	
F15	0.4170 (2)	0.4340 (2)	0.35735 (18)	0.0835 (7)	
F16	0.6211 (2)	0.38520 (17)	0.42687 (17)	0.0755 (7)	
F22	1.2472 (2)	0.4817 (3)	0.20934 (17)	0.0965 (9)	
F23	1.2652 (3)	0.4141 (4)	0.05944 (19)	0.1436 (15)	
F24	1.1102 (3)	0.2599 (3)	0.0439 (2)	0.1320 (13)	
F25	0.9324 (3)	0.1744 (2)	0.18009 (19)	0.1020 (9)	
F26	0.9066 (2)	0.24546 (19)	0.32861 (15)	0.0752 (7)	
F32	0.3270 (2)	1.1741 (2)	0.24713 (18)	0.0853 (8)	
F33	0.3367 (3)	1.2075 (2)	0.4066 (2)	0.1137 (11)	
F34	0.5380 (4)	1.1420 (3)	0.48502 (19)	0.1228 (12)	
F35	0.7289 (3)	1.0388 (3)	0.4018 (2)	0.1189 (11)	
F36	0.7184 (2)	1.0020 (2)	0.24348 (18)	0.0859 (8)	
F42	0.4421 (2)	0.58974 (19)	0.06455 (16)	0.0742 (7)	
F43	0.5080 (3)	0.3749 (2)	0.13442 (19)	0.0936 (9)	
F44	0.7054 (3)	0.3328 (2)	0.2180 (2)	0.1089 (10)	
F45	0.8342 (3)	0.5075 (2)	0.2356 (2)	0.1015 (9)	
F46	0.7659 (2)	0.7222 (2)	0.16930 (18)	0.0788 (7)	
C1	0.7799 (3)	0.5529 (3)	0.4461 (2)	0.0403 (7)	
C2	1.0579 (3)	0.4126 (3)	0.3560 (2)	0.0427 (8)	
C3	0.5132 (3)	1.0596 (3)	0.1533 (2)	0.0508 (9)	
C4	0.5663 (3)	0.7837 (3)	0.0740 (2)	0.0474 (8)	
C11	0.6619 (3)	0.5797 (3)	0.4104 (2)	0.0415 (8)	
C12	0.6210 (3)	0.6906 (3)	0.3807 (2)	0.0463 (8)	
C13	0.5158 (3)	0.7157 (3)	0.3439 (3)	0.0536 (9)	
C14	0.4466 (3)	0.6292 (3)	0.3362 (2)	0.0549 (9)	
C15	0.4835 (3)	0.5191 (3)	0.3652 (2)	0.0553 (9)	
C16	0.5887 (3)	0.4949 (3)	0.4021 (2)	0.0500 (9)	
C21	1.0765 (3)	0.3675 (3)	0.2753 (2)	0.0462 (8)	
C22	1.1659 (4)	0.4077 (4)	0.2043 (3)	0.0645 (11)	
C23	1.1781 (4)	0.3726 (5)	0.1276 (3)	0.0801 (14)	
C24	1.1001 (4)	0.2942 (4)	0.1191 (3)	0.0815 (14)	
C25	1.0104 (4)	0.2503 (4)	0.1882 (3)	0.0677 (11)	
C26	0.9990 (4)	0.2862 (3)	0.2652 (2)	0.0532 (9)	
C31	0.5223 (4)	1.0865 (3)	0.2391 (2)	0.0531 (9)	
C32	0.4272 (4)	1.1393 (3)	0.2834 (3)	0.0656 (11)	
C33	0.4316 (5)	1.1583 (4)	0.3654 (3)	0.0809 (14)	
C34	0.5336 (6)	1.1253 (4)	0.4042 (3)	0.0870 (15)	
C35	0.6308 (5)	1.0742 (4)	0.3626 (3)	0.0790 (13)	
C36	0.6232 (4)	1.0558 (3)	0.2805 (3)	0.0630 (11)	
C41	0.6021 (3)	0.6640 (3)	0.1133 (2)	0.0454 (8)	
C42	0.5378 (3)	0.5724 (3)	0.1074 (2)	0.0524 (9)	
C43	0.5718 (4)	0.4619 (3)	0.1413 (3)	0.0620 (10)	
C44	0.6713 (4)	0.4407 (3)	0.1836 (3)	0.0702 (12)	
C45	0.7374 (4)	0.5291 (4)	0.1916 (3)	0.0670 (11)	
C46	0.7020 (3)	0.6383 (3)	0.1571 (3)	0.0550 (9)	
O1S	0.9210 (13)	0.2122 (11)	0.6115 (10)	0.099 (5)	0.50
C11S	1.0060 (11)	0.1307 (9)	0.6307 (8)	0.087 (3)*	0.50
H11A	1.0176	0.1200	0.6919	0.104*	0.50
H11B	1.0831	0.1539	0.5931	0.104*	0.50
C12S	0.9670 (10)	0.0177 (9)	0.6161 (8)	0.086 (3)*	0.50
H12A	1.0268	−0.0096	0.5735	0.103*	0.50
H12B	0.9545	−0.0406	0.6714	0.103*	0.50
C13S	0.8504 (18)	0.0515 (17)	0.5810 (13)	0.150 (8)*	0.50
H13A	0.7873	−0.0031	0.6109	0.181*	0.50
H13B	0.8610	0.0565	0.5178	0.181*	0.50
C14S	0.8182 (18)	0.1656 (18)	0.5997 (15)	0.133 (10)	0.50
H14A	0.7841	0.2148	0.5504	0.160*	0.50
H14B	0.7576	0.1574	0.6526	0.160*	0.50
O2S	0.9156 (12)	0.2129 (8)	0.6036 (6)	0.051 (3)	0.50
C21S	0.8251 (15)	0.1678 (11)	0.5661 (11)	0.069 (4)	0.50
H21A	0.8520	0.1719	0.5037	0.082*	0.50
H21B	0.7481	0.2091	0.5728	0.082*	0.50
C22S	0.8129 (14)	0.0451 (9)	0.6193 (10)	0.099 (4)	0.50
H22A	0.7442	0.0370	0.6670	0.119*	0.50
H22B	0.8017	−0.0048	0.5817	0.119*	0.50
C23S	0.9201 (14)	0.0206 (12)	0.6521 (10)	0.117 (5)*	0.50
H23A	0.9524	−0.0528	0.6440	0.141*	0.50
H23B	0.9079	0.0183	0.7150	0.141*	0.50
C24S	1.0179 (9)	0.1258 (8)	0.5949 (7)	0.068 (3)*	0.50
H24A	1.0853	0.1333	0.6251	0.082*	0.50
H24B	1.0469	0.1218	0.5342	0.082*	0.50
O3S	0.1960 (4)	0.8917 (3)	0.0690 (2)	0.0499 (18)	0.50
C31S	0.1619 (5)	0.8109 (5)	0.0306 (4)	0.117 (4)*	0.50
H31A	0.2335	0.7700	0.0093	0.140*	0.50
H31B	0.1214	0.8471	−0.0196	0.140*	0.50
C32S	0.0765 (8)	0.7290 (5)	0.0986 (7)	0.141 (5)*	0.50
H32A	0.1196	0.6645	0.1311	0.169*	0.50
H32B	0.0138	0.7022	0.0726	0.169*	0.50
C33S	0.0260 (3)	0.8101 (9)	0.1551 (9)	0.277 (16)	0.50
H33A	−0.0099	0.7698	0.2143	0.332*	0.50
H33B	−0.0338	0.8628	0.1280	0.332*	0.50
C34S	0.1417 (7)	0.8695 (10)	0.1559 (3)	0.111 (4)*	0.50
H34A	0.1236	0.9394	0.1754	0.133*	0.50
H34B	0.1932	0.8206	0.1943	0.133*	0.50
O4S	0.1905 (13)	0.9120 (11)	0.0840 (10)	0.178 (6)	0.50
C41S	0.0973 (14)	0.9482 (13)	0.0497 (10)	0.139 (5)*	0.50
H41A	0.1143	0.9505	−0.0131	0.167*	0.50
H41B	0.0796	1.0255	0.0555	0.167*	0.50
C42S	−0.0081 (12)	0.8803 (16)	0.0888 (14)	0.206 (10)	0.50
H42A	−0.0421	0.8512	0.0458	0.248*	0.50
H42B	−0.0709	0.9204	0.1204	0.248*	0.50
C43S	0.0749 (17)	0.7609 (15)	0.1692 (12)	0.152 (6)*	0.50
H43A	0.0812	0.7800	0.2246	0.183*	0.50
H43C	0.0389	0.6865	0.1806	0.183*	0.50
C44S	0.1788 (19)	0.7709 (16)	0.1144 (14)	0.197 (8)*	0.50
H44C	0.1744	0.7391	0.0642	0.236*	0.50
H44A	0.2461	0.7352	0.1441	0.236*	0.50

### Atomic displacement parameters (Å^2^)

**Table:**

	*U*^11^	*U*^22^	*U*^33^	*U*^12^	*U*^13^	*U*^23^
Mo1	0.03771 (16)	0.03352 (14)	0.03634 (15)	−0.00466 (11)	−0.00365 (11)	−0.00546 (11)
Mo2	0.03164 (15)	0.04565 (17)	0.05316 (19)	−0.00679 (12)	−0.00261 (13)	−0.00081 (14)
O1	0.0402 (12)	0.0391 (11)	0.0445 (13)	−0.0045 (10)	−0.0066 (10)	−0.0033 (10)
O2	0.0395 (12)	0.0380 (11)	0.0459 (12)	−0.0067 (9)	−0.0062 (10)	−0.0037 (10)
O3	0.0504 (13)	0.0417 (12)	0.0375 (12)	−0.0060 (10)	0.0000 (10)	−0.0068 (10)
O4	0.0522 (14)	0.0386 (11)	0.0415 (12)	−0.0039 (10)	−0.0043 (10)	−0.0101 (10)
O5	0.0407 (13)	0.0553 (14)	0.0587 (15)	−0.0092 (11)	−0.0058 (11)	−0.0067 (12)
O6	0.0408 (13)	0.0548 (14)	0.0542 (14)	−0.0079 (11)	−0.0008 (11)	−0.0045 (11)
O7	0.0344 (12)	0.0490 (13)	0.0594 (15)	−0.0054 (10)	−0.0017 (11)	0.0000 (11)
O8	0.0360 (13)	0.0464 (13)	0.0587 (15)	−0.0062 (10)	−0.0027 (11)	0.0001 (11)
F12	0.0596 (13)	0.0445 (11)	0.1041 (18)	−0.0037 (10)	−0.0266 (12)	−0.0145 (12)
F13	0.0676 (15)	0.0561 (13)	0.121 (2)	0.0046 (11)	−0.0387 (15)	0.0045 (13)
F14	0.0577 (14)	0.0952 (17)	0.0857 (17)	−0.0070 (12)	−0.0365 (13)	−0.0054 (14)
F15	0.0716 (15)	0.0745 (15)	0.115 (2)	−0.0246 (12)	−0.0376 (14)	−0.0216 (14)
F16	0.0752 (15)	0.0434 (11)	0.1131 (19)	−0.0069 (11)	−0.0370 (14)	−0.0131 (12)
F22	0.0740 (17)	0.152 (2)	0.0734 (16)	−0.0499 (17)	0.0211 (13)	−0.0567 (17)
F23	0.111 (2)	0.257 (4)	0.0800 (19)	−0.073 (3)	0.0390 (17)	−0.093 (2)
F24	0.121 (3)	0.215 (3)	0.096 (2)	−0.021 (2)	−0.0015 (18)	−0.112 (2)
F25	0.117 (2)	0.109 (2)	0.103 (2)	−0.0284 (18)	−0.0212 (17)	−0.0615 (17)
F26	0.0893 (17)	0.0734 (14)	0.0640 (14)	−0.0309 (13)	−0.0028 (13)	−0.0182 (12)
F32	0.0765 (17)	0.0821 (16)	0.0932 (19)	0.0154 (14)	−0.0082 (15)	−0.0199 (15)
F33	0.139 (3)	0.0940 (19)	0.108 (2)	−0.0160 (19)	0.027 (2)	−0.0506 (18)
F34	0.188 (3)	0.118 (2)	0.0710 (18)	−0.057 (2)	−0.013 (2)	−0.0309 (17)
F35	0.132 (3)	0.130 (3)	0.106 (2)	−0.027 (2)	−0.069 (2)	−0.0113 (19)
F36	0.0675 (16)	0.0977 (18)	0.0936 (19)	0.0037 (14)	−0.0282 (14)	−0.0162 (15)
F42	0.0771 (16)	0.0681 (14)	0.0807 (16)	−0.0129 (12)	−0.0302 (13)	−0.0097 (12)
F43	0.118 (2)	0.0549 (13)	0.108 (2)	−0.0211 (14)	−0.0214 (17)	−0.0135 (14)
F44	0.128 (3)	0.0550 (14)	0.130 (3)	0.0181 (15)	−0.031 (2)	0.0057 (15)
F45	0.0787 (18)	0.0917 (18)	0.129 (2)	0.0202 (15)	−0.0486 (17)	−0.0009 (17)
F46	0.0626 (14)	0.0711 (14)	0.1058 (19)	−0.0071 (12)	−0.0356 (13)	−0.0109 (14)
C1	0.0410 (18)	0.0435 (17)	0.0369 (17)	−0.0028 (14)	−0.0043 (14)	−0.0113 (15)
C2	0.0393 (18)	0.0473 (19)	0.0424 (18)	0.0021 (14)	−0.0078 (14)	−0.0123 (15)
C3	0.049 (2)	0.0427 (18)	0.055 (2)	−0.0031 (16)	−0.0067 (17)	0.0001 (16)
C4	0.043 (2)	0.0466 (18)	0.048 (2)	−0.0027 (16)	−0.0044 (16)	−0.0037 (16)
C11	0.0393 (18)	0.0448 (17)	0.0389 (17)	−0.0051 (14)	−0.0049 (14)	−0.0070 (14)
C12	0.0398 (18)	0.0452 (18)	0.053 (2)	−0.0065 (15)	−0.0086 (15)	−0.0090 (16)
C13	0.048 (2)	0.048 (2)	0.060 (2)	0.0011 (16)	−0.0098 (17)	−0.0026 (17)
C14	0.0393 (19)	0.072 (2)	0.052 (2)	−0.0047 (18)	−0.0135 (16)	−0.0082 (19)
C15	0.052 (2)	0.058 (2)	0.058 (2)	−0.0193 (18)	−0.0111 (18)	−0.0110 (18)
C16	0.051 (2)	0.0441 (19)	0.055 (2)	−0.0055 (16)	−0.0133 (17)	−0.0081 (16)
C21	0.0435 (19)	0.0516 (19)	0.0464 (19)	0.0069 (15)	−0.0084 (15)	−0.0179 (16)
C22	0.052 (2)	0.089 (3)	0.059 (2)	−0.010 (2)	−0.0025 (19)	−0.033 (2)
C23	0.060 (3)	0.129 (4)	0.059 (3)	−0.016 (3)	0.008 (2)	−0.045 (3)
C24	0.076 (3)	0.119 (4)	0.066 (3)	0.005 (3)	−0.013 (2)	−0.053 (3)
C25	0.071 (3)	0.073 (3)	0.073 (3)	0.001 (2)	−0.023 (2)	−0.036 (2)
C26	0.061 (2)	0.051 (2)	0.052 (2)	0.0050 (17)	−0.0126 (18)	−0.0203 (17)
C31	0.061 (2)	0.0406 (18)	0.053 (2)	−0.0109 (17)	−0.0088 (18)	−0.0005 (16)
C32	0.076 (3)	0.049 (2)	0.067 (3)	−0.010 (2)	−0.007 (2)	−0.006 (2)
C33	0.107 (4)	0.052 (2)	0.078 (3)	−0.022 (2)	0.012 (3)	−0.018 (2)
C34	0.126 (5)	0.071 (3)	0.064 (3)	−0.043 (3)	−0.014 (3)	−0.009 (2)
C35	0.095 (4)	0.070 (3)	0.074 (3)	−0.030 (3)	−0.030 (3)	−0.003 (2)
C36	0.067 (3)	0.053 (2)	0.069 (3)	−0.0145 (19)	−0.016 (2)	−0.007 (2)
C41	0.0420 (19)	0.0474 (18)	0.0417 (18)	−0.0033 (15)	0.0012 (15)	−0.0056 (15)
C42	0.049 (2)	0.058 (2)	0.046 (2)	−0.0034 (17)	−0.0040 (17)	−0.0061 (17)
C43	0.072 (3)	0.051 (2)	0.059 (2)	−0.011 (2)	−0.004 (2)	−0.0072 (19)
C44	0.077 (3)	0.049 (2)	0.073 (3)	0.010 (2)	−0.006 (2)	0.000 (2)
C45	0.059 (3)	0.069 (3)	0.066 (3)	0.011 (2)	−0.015 (2)	−0.002 (2)
C46	0.044 (2)	0.057 (2)	0.059 (2)	−0.0016 (17)	−0.0048 (17)	−0.0075 (18)
O1S	0.059 (7)	0.067 (7)	0.170 (12)	0.000 (6)	−0.002 (7)	−0.037 (7)
C14S	0.064 (9)	0.140 (15)	0.19 (2)	0.018 (9)	−0.017 (11)	−0.031 (14)
O2S	0.082 (8)	0.025 (4)	0.039 (4)	−0.014 (4)	−0.008 (4)	0.006 (3)
C21S	0.069 (7)	0.044 (5)	0.093 (8)	−0.036 (5)	−0.007 (6)	−0.013 (5)
C22S	0.143 (11)	0.046 (5)	0.110 (10)	−0.057 (6)	−0.011 (9)	−0.012 (6)
O3S	0.018 (2)	0.052 (3)	0.068 (4)	−0.017 (2)	0.020 (3)	−0.007 (3)
C33S	0.061 (9)	0.35 (3)	0.29 (3)	−0.089 (14)	−0.043 (12)	0.19 (2)
O4S	0.204 (12)	0.115 (8)	0.237 (14)	−0.066 (8)	−0.133 (11)	−0.007 (9)
C42S	0.068 (9)	0.219 (18)	0.30 (2)	−0.051 (10)	−0.070 (12)	0.044 (17)

### Geometric parameters (Å, °)

**Table:**

Mo1—O3^i^	2.096 (2)	C31—C32	1.384 (6)
Mo1—Mo1^i^	2.1090 (7)	C32—C33	1.375 (6)
Mo1—O1^i^	2.111 (2)	C33—C34	1.367 (7)
Mo1—O2	2.116 (2)	C34—C35	1.369 (7)
Mo1—O4	2.118 (2)	C35—C36	1.381 (6)
Mo1—O2S	2.530 (9)	C41—C46	1.381 (5)
Mo1—O1S	2.543 (12)	C41—C42	1.381 (5)
Mo2—O6	2.106 (3)	C42—C43	1.369 (5)
Mo2—O7^ii^	2.108 (2)	C43—C44	1.361 (6)
Mo2—Mo2^ii^	2.1101 (8)	C44—C45	1.372 (6)
Mo2—O5	2.113 (3)	C45—C46	1.362 (5)
Mo2—O8	2.118 (2)	O1S—C11S	1.341 (17)
Mo2—O4S	2.544 (14)	O1S—C14S	1.36 (3)
Mo2—O3S	2.552 (4)	C11S—C12S	1.538 (15)
O1—C1	1.264 (4)	C11S—H11A	0.9700
O1—Mo1^i^	2.111 (2)	C11S—H11B	0.9700
O2—C1	1.263 (4)	C12S—C13S	1.49 (2)
O3—C2	1.265 (4)	C12S—H12A	0.9700
O3—Mo1^i^	2.096 (2)	C12S—H12B	0.9700
O4—C2	1.265 (4)	C13S—C14S	1.50 (3)
O5—C3^ii^	1.265 (4)	C13S—H13A	0.9700
O6—C3	1.272 (4)	C13S—H13B	0.9700
O7—C4	1.263 (4)	C14S—H14A	0.9700
O7—Mo2^ii^	2.108 (2)	C14S—H14B	0.9700
O8—C4	1.261 (4)	O2S—C21S	1.44 (2)
F12—C12	1.336 (4)	O2S—C24S	1.520 (15)
F13—C13	1.328 (4)	C21S—C22S	1.516 (17)
F14—C14	1.334 (4)	C21S—H21A	0.9700
F15—C15	1.342 (4)	C21S—H21B	0.9700
F16—C16	1.335 (4)	C22S—C23S	1.363 (19)
F22—C22	1.330 (5)	C22S—H22A	0.9700
F23—C23	1.337 (5)	C22S—H22B	0.9700
F24—C24	1.335 (5)	C23S—C24S	1.697 (17)
F25—C25	1.339 (5)	C23S—H23A	0.9700
F26—C26	1.332 (4)	C23S—H23B	0.9700
F32—C32	1.332 (5)	C24S—H24A	0.9700
F33—C33	1.336 (5)	C24S—H24B	0.9700
F34—C34	1.345 (5)	O3S—C34S	1.3700 (11)
F35—C35	1.336 (6)	O3S—C31S	1.3703 (11)
F36—C36	1.339 (5)	C31S—C32S	1.5097 (11)
F42—C42	1.330 (4)	C31S—H31A	0.9700
F43—C43	1.337 (4)	C31S—H31B	0.9700
F44—C44	1.344 (4)	C32S—C33S	1.5098 (11)
F45—C45	1.350 (5)	C32S—H32A	0.9700
F46—C46	1.339 (4)	C32S—H32B	0.9700
C1—C11	1.496 (4)	C33S—C34S	1.5101 (11)
C2—C21	1.485 (5)	C33S—H33A	0.9700
C3—O5^ii^	1.265 (4)	C33S—H33B	0.9700
C3—C31	1.486 (5)	C34S—H34A	0.9700
C4—C41	1.488 (5)	C34S—H34B	0.9700
C11—C12	1.385 (5)	O4S—C41S	1.251 (18)
C11—C16	1.389 (4)	O4S—C44S	1.66 (2)
C12—C13	1.373 (5)	C41S—C42S	1.431 (18)
C13—C14	1.373 (5)	C41S—H41A	0.9700
C14—C15	1.362 (5)	C41S—H41B	0.9700
C15—C16	1.372 (5)	C42S—C43S	1.97 (2)
C21—C22	1.377 (5)	C42S—H42A	0.9700
C21—C26	1.397 (5)	C42S—H42B	0.9700
C22—C23	1.361 (6)	C43S—C44S	1.32 (2)
C23—C24	1.365 (6)	C43S—H43A	0.9700
C24—C25	1.370 (6)	C43S—H43C	0.9700
C25—C26	1.371 (5)	C44S—H44C	0.9700
C31—C36	1.375 (6)	C44S—H44A	0.9700
			
O3^i^—Mo1—Mo1^i^	92.91 (6)	C43—C42—C41	122.4 (4)
O3^i^—Mo1—O1^i^	87.28 (9)	F43—C43—C44	119.7 (4)
Mo1^i^—Mo1—O1^i^	91.21 (6)	F43—C43—C42	121.1 (4)
O3^i^—Mo1—O2	91.98 (9)	C44—C43—C42	119.2 (4)
Mo1^i^—Mo1—O2	92.05 (6)	F44—C44—C43	120.1 (4)
O1^i^—Mo1—O2	176.69 (8)	F44—C44—C45	119.5 (4)
O3^i^—Mo1—O4	176.72 (8)	C43—C44—C45	120.4 (4)
Mo1^i^—Mo1—O4	90.36 (6)	F45—C45—C46	120.6 (4)
O1^i^—Mo1—O4	92.37 (9)	F45—C45—C44	120.1 (4)
O2—Mo1—O4	88.19 (9)	C46—C45—C44	119.3 (4)
O3^i^—Mo1—O2S	100.2 (2)	F46—C46—C45	117.6 (4)
Mo1^i^—Mo1—O2S	166.1 (2)	F46—C46—C41	120.1 (3)
O1^i^—Mo1—O2S	84.8 (3)	C45—C46—C41	122.4 (4)
O2—Mo1—O2S	92.1 (3)	C11S—O1S—C14S	110.0 (14)
O4—Mo1—O2S	76.5 (2)	C11S—O1S—Mo1	124.9 (9)
O3^i^—Mo1—O1S	97.8 (3)	C14S—O1S—Mo1	119.8 (12)
Mo1^i^—Mo1—O1S	167.2 (3)	O1S—C11S—C12S	110.6 (11)
O1^i^—Mo1—O1S	82.4 (3)	O1S—C11S—H11A	109.5
O2—Mo1—O1S	94.5 (3)	C12S—C11S—H11A	109.5
O4—Mo1—O1S	78.9 (3)	O1S—C11S—H11B	109.5
O2S—Mo1—O1S	3.3 (6)	C12S—C11S—H11B	109.5
O6—Mo2—O7^ii^	89.90 (10)	H11A—C11S—H11B	108.1
O6—Mo2—Mo2^ii^	92.15 (7)	C13S—C12S—C11S	101.7 (11)
O7^ii^—Mo2—Mo2^ii^	92.32 (6)	C13S—C12S—H12A	111.4
O6—Mo2—O5	176.67 (9)	C11S—C12S—H12A	111.4
O7^ii^—Mo2—O5	90.01 (10)	C13S—C12S—H12B	111.4
Mo2^ii^—Mo2—O5	91.18 (6)	C11S—C12S—H12B	111.4
O6—Mo2—O8	89.85 (10)	H12A—C12S—H12B	109.3
O7^ii^—Mo2—O8	176.77 (8)	C12S—C13S—C14S	104.9 (16)
Mo2^ii^—Mo2—O8	90.91 (6)	C12S—C13S—H13A	110.8
O5—Mo2—O8	90.05 (9)	C14S—C13S—H13A	110.8
O6—Mo2—O4S	87.9 (3)	C12S—C13S—H13B	110.8
O7^ii^—Mo2—O4S	93.5 (3)	C14S—C13S—H13B	110.8
Mo2^ii^—Mo2—O4S	174.1 (3)	H13A—C13S—H13B	108.8
O5—Mo2—O4S	88.8 (3)	O1S—C14S—C13S	108.7 (16)
O8—Mo2—O4S	83.2 (3)	O1S—C14S—H14A	110.0
O6—Mo2—O3S	95.48 (11)	C13S—C14S—H14A	110.0
O7^ii^—Mo2—O3S	97.35 (11)	O1S—C14S—H14B	110.0
Mo2^ii^—Mo2—O3S	167.68 (9)	C13S—C14S—H14B	110.0
O5—Mo2—O3S	81.23 (11)	H14A—C14S—H14B	108.3
O8—Mo2—O3S	79.47 (11)	C21S—O2S—C24S	99.8 (10)
O4S—Mo2—O3S	8.5 (4)	C21S—O2S—Mo1	116.3 (8)
C1—O1—Mo1^i^	116.9 (2)	C24S—O2S—Mo1	117.6 (7)
C1—O2—Mo1	115.87 (19)	O2S—C21S—C22S	103.9 (12)
C2—O3—Mo1^i^	115.9 (2)	O2S—C21S—H21A	111.0
C2—O4—Mo1	117.2 (2)	C22S—C21S—H21A	111.0
C3^ii^—O5—Mo2	117.1 (2)	O2S—C21S—H21B	111.0
C3—O6—Mo2	116.3 (2)	C22S—C21S—H21B	111.0
C4—O7—Mo2^ii^	115.9 (2)	H21A—C21S—H21B	109.0
C4—O8—Mo2	116.8 (2)	C23S—C22S—C21S	104.4 (11)
O2—C1—O1	123.8 (3)	C23S—C22S—H22A	110.9
O2—C1—C11	118.4 (3)	C21S—C22S—H22A	110.9
O1—C1—C11	117.8 (3)	C23S—C22S—H22B	110.9
O3—C2—O4	123.5 (3)	C21S—C22S—H22B	110.9
O3—C2—C21	118.1 (3)	H22A—C22S—H22B	108.9
O4—C2—C21	118.4 (3)	C22S—C23S—C24S	106.6 (10)
O5^ii^—C3—O6	123.2 (3)	C22S—C23S—H23A	110.4
O5^ii^—C3—C31	118.5 (3)	C24S—C23S—H23A	110.4
O6—C3—C31	118.3 (3)	C22S—C23S—H23B	110.4
O8—C4—O7	124.0 (3)	C24S—C23S—H23B	110.4
O8—C4—C41	118.1 (3)	H23A—C23S—H23B	108.6
O7—C4—C41	117.9 (3)	O2S—C24S—C23S	89.3 (8)
C12—C11—C16	115.8 (3)	O2S—C24S—H24A	113.8
C12—C11—C1	122.2 (3)	C23S—C24S—H24A	113.8
C16—C11—C1	121.9 (3)	O2S—C24S—H24B	113.8
F12—C12—C13	117.1 (3)	C23S—C24S—H24B	113.8
F12—C12—C11	120.6 (3)	H24A—C24S—H24B	111.0
C13—C12—C11	122.4 (3)	C34S—O3S—C31S	109.1 (5)
F13—C13—C14	119.6 (3)	C34S—O3S—Mo2	120.4 (3)
F13—C13—C12	120.5 (3)	C31S—O3S—Mo2	121.2 (3)
C14—C13—C12	119.9 (3)	O3S—C31S—C32S	108.9 (5)
F14—C14—C15	120.4 (3)	O3S—C31S—H31A	109.9
F14—C14—C13	120.2 (3)	C32S—C31S—H31A	109.9
C15—C14—C13	119.4 (3)	O3S—C31S—H31B	109.9
F15—C15—C14	119.8 (3)	C32S—C31S—H31B	109.9
F15—C15—C16	119.9 (3)	H31A—C31S—H31B	108.3
C14—C15—C16	120.3 (3)	C31S—C32S—C33S	97.5 (6)
F16—C16—C15	117.0 (3)	C31S—C32S—H32A	112.3
F16—C16—C11	120.7 (3)	C33S—C32S—H32A	112.3
C15—C16—C11	122.2 (3)	C31S—C32S—H32B	112.3
C22—C21—C26	116.3 (3)	C33S—C32S—H32B	112.3
C22—C21—C2	122.2 (3)	H32A—C32S—H32B	109.9
C26—C21—C2	121.4 (3)	C32S—C33S—C34S	99.9 (6)
F22—C22—C23	117.0 (4)	C32S—C33S—H33A	111.8
F22—C22—C21	120.4 (3)	C34S—C33S—H33A	111.8
C23—C22—C21	122.6 (4)	C32S—C33S—H33B	111.8
F23—C23—C22	121.2 (4)	C34S—C33S—H33B	111.8
F23—C23—C24	118.9 (4)	H33A—C33S—H33B	109.5
C22—C23—C24	119.9 (4)	O3S—C34S—C33S	103.0 (6)
F24—C24—C23	120.5 (4)	O3S—C34S—H34A	111.2
F24—C24—C25	119.6 (4)	C33S—C34S—H34A	111.2
C23—C24—C25	119.9 (4)	O3S—C34S—H34B	111.2
F25—C25—C24	120.3 (4)	C33S—C34S—H34B	111.2
F25—C25—C26	120.0 (4)	H34A—C34S—H34B	109.1
C24—C25—C26	119.7 (4)	C41S—O4S—C44S	105.8 (13)
F26—C26—C25	117.4 (3)	C41S—O4S—Mo2	125.7 (12)
F26—C26—C21	120.8 (3)	C44S—O4S—Mo2	113.8 (11)
C25—C26—C21	121.6 (4)	O4S—C41S—C42S	113.5 (15)
C36—C31—C32	116.5 (4)	O4S—C41S—H41A	108.9
C36—C31—C3	121.8 (4)	C42S—C41S—H41A	108.9
C32—C31—C3	121.6 (4)	O4S—C41S—H41B	108.9
F32—C32—C33	117.3 (4)	C42S—C41S—H41B	108.9
F32—C32—C31	120.5 (4)	H41A—C41S—H41B	107.7
C33—C32—C31	122.2 (5)	C41S—C42S—C43S	96.4 (12)
F33—C33—C34	120.7 (5)	C41S—C42S—H42A	112.5
F33—C33—C32	120.2 (5)	C43S—C42S—H42A	112.5
C34—C33—C32	119.1 (5)	C41S—C42S—H42B	112.5
F34—C34—C33	119.6 (6)	C43S—C42S—H42B	112.5
F34—C34—C35	119.4 (6)	H42A—C42S—H42B	110.0
C33—C34—C35	121.0 (5)	C44S—C43S—C42S	93.6 (15)
F35—C35—C34	121.0 (5)	C44S—C43S—H43A	113.0
F35—C35—C36	120.5 (5)	C42S—C43S—H43A	113.0
C34—C35—C36	118.4 (5)	C44S—C43S—H43C	113.0
F36—C36—C31	120.6 (4)	C42S—C43S—H43C	113.0
F36—C36—C35	116.6 (4)	H43A—C43S—H43C	110.4
C31—C36—C35	122.8 (4)	C43S—C44S—O4S	100.4 (16)
C46—C41—C42	116.3 (3)	C43S—C44S—H44C	111.7
C46—C41—C4	121.8 (3)	O4S—C44S—H44C	111.7
C42—C41—C4	121.9 (3)	C43S—C44S—H44A	111.7
F42—C42—C43	117.4 (3)	O4S—C44S—H44A	111.7
F42—C42—C41	120.1 (3)	H44C—C44S—H44A	109.5
			
O1—C1—C11—C12	−28.1 (5)	O5^ii^—C3—C31—C36	−39.1 (5)
O2—C1—C11—C16	−29.3 (5)	O6—C3—C31—C32	−37.7 (5)
O3—C2—C21—C22	30.4 (5)	O7—C4—C41—C46	40.9 (5)
O4—C2—C21—C26	31.6 (5)	O8—C4—C41—C42	41.7 (5)

Symmetry codes: (i) −*x*+2, −*y*+1, −*z*+1; (ii) −*x*+1, −*y*+2, −*z*.
